# Estimated date of delivery in Chihuahua breed bitches, based on embryo-fetal biometry, assessed by ultrasonography

**DOI:** 10.1590/1984-3143-AR2020-0037

**Published:** 2020-08-04

**Authors:** Catharina de Albuquerque Vieira, Rodrigo Freitas Bittencourt, Carmo Emanuel Almeida Biscarde, Maíra Planzo Fernandes, Alessandro Bitencourt Nascimento, Elton Amorim Romão, Isabella de Matos Brandão Carneiro, Mariana Alves de Andrade Silva, Renata Oliveira Barreto, Marcus Vinícius Galvão Loiola

**Affiliations:** 1 Setor de Reprodução Animal e Obstetrícia, Hospital Veterinário, Escola de Medicina Veterinária e Zootecnia, Universidade Federal da Bahia, Salvador, BA, Brasil

**Keywords:** canine, gestational age, pregnancy, ultrasound

## Abstract

Due to the peculiarities of the reproductive cycle of the female dog, which makes it difficult to accurately ascertain the date of conception, it may be challenging to precisely estimate the gestational age in bitches. Using fetal measurements obtained by ultrasound, it is possible to estimate the gestational age in dogs; however, due to the differences in size of the various breeds, such estimates may have a significant standard deviation, which represents less accuracy when specifying the date of birth. The purpose of this study was to evaluate pregnant female Chihuahuas, establishing relations between the fetal dimensions measured by ultrasound and the remaining time until delivery. Using 13 pregnant female Chihuahuas, weekly ultrasound assessments and measurements were performed, of the inner chorionic cavity, cranial-caudal length, biparietal diameter, abdominal diameter and thoracic diameter. Such parameters were retroactively correlated to the date of delivery, and linear regressions were established between the gestational measurements and remaining days until delivery. The fetal measurement presenting the highest correlation (*r* = 0.99; P<0.0001) and reliability (R^2^ = 0.98, *P<*0.0001) with the probable date of delivery was the biparietal diameter, a measurement that can be easily and safely obtained and, when applying a specific formula (Y = -15.46X1 + 38.72), can accurately predict the date of delivery in Chihuahua female dogs.

## Introduction

In the canine species, the time elapsed from the date of mating does not necessarily coincide with the gestational time. This is due to the long period of sexual receptivity of the female bitch, such that the bitch may stand to be bred either long before of well after ovulation occurs. Additionally, variation in length between mating and delivery is dependent on the prolonged viability of the sperm in the reproductive tract, which leads to a variation in the estimate of the gestational duration from 57-68 days after copulation (Concannon and Lein, 1989).

In fact, parturition occurs 65 ± 1 days after LH peak and 63 ± 1 days after ovulation (Concannon et al., 1983). However, detection of LH peak and the ultrasonographic follow-up of ovulation are unpractical because of the requirement of daily examinations (Hase et al., 2000). Therefore, in order to plan and monitor the deliveries, a more practical method for establishing the gestational age is required.

B-mode ultrasonography is a semiological means of relatively low cost, is non-invasive, harmless to the bitch and puppies, and provides valuable information about the fetal development (Papp and Fekete, 2003). With ultrasonography, it is possible to estimate the gestational age, by means of formulas that relate fetal measurements to the gestational period, in addition to assessing the fetal viability (Beccaglia et al., 2016).

Ultrasonographic assessment of the gestational age in female dogs is somewhat limited by the great variability in size and body conformation of different breeds of dogs. Therefore, there have been attempts to establish more specific formulas for different breeds, sizes and cranial conformations, to better assist clinicians in monitoring the gestational age of female dogs (Camargo et al., 2011).

Several authors have studied the relationship between fetal biometrics and the development of the pregnancy in different breeds of different sizes, such as the Boxer (Silva et al., 2007), Yorkshire Terrier (Jabin et al., 2007), American Cocker Spaniel and Chow-Chow (Melo et al., 2006), Rottweiler (Teixeira et al., 2009), German Shepherd (Groppetti et al., 2015; Cecchetto et al., 2017; Socha and Janowski, 2017), observing differences between the biometric formulas used for each size or breed of the studied dogs.

In the authors clinical experience, was observed that the formula used to estimate the gestational age (*GA* = biparietal diameter × 15 + 20), described by Nyland and Mattoon (2002), for all breeds of dogs, did not correspond to the correct gestational age at the end of the pregnancy of female Chihuahua dogs.

The Chihuahua breed is widely distributed globally and is considered the smallest dog breed in the world (FCI, 2010), with a relatively large skull (Traas, 2008) and a high risk of dystocia (Gaudet, 1985; Bergström et al., 2006; O’Neill et al., 2017). Due to these specific biometric traits, it is difficult to apply general formulas to precisely determine the pregnancy duration, which is necessary to determine due date and make plans for possible interventions in cases of dystocia.

To our knowledge, there are no currently available published studies that use fetal biometry to estimate the gestational age specific for dogs of the Chihuahua breed. Thus, the purpose of this study was to monitor pregnant Chihuahuas and to establish formulas that will relate fetal measurements to the gestational age, and determine the expected date of delivery.

## Methods

The experiment was conducted in the Animal Reproduction and Veterinary Obstetrics Department of the Hospital of Veterinary Medicine, School of Veterinary Medicine and Animal Science of the Federal University. The study was approved by the local Ethics Committee on Animal Use (CEUA), under protocol EMEVZ nº. 10/2017.

Thirteen pregnant Chihuahuas were used (eight long-haired and five short-haired), five primiparous and eight multiparous, between 1-3 years old and a body weight between 2-4 kg. The females belonged to both commercial kennels and hobby breeders, and were mated with males of the same breed using natural mating or artificial insemination techniques, according to the reproductive program of each breeder.

To perform the obstetric ultrasound exam, the dogs were positioned and restrained in dorsal decubitus on a padded surface specific for the purpose, without sedation, and the females were prepared with abdominal trichotomy from the xiphoid cartilage to the pubis, extending laterally to the renal region.

A portable ultrasound device (Z5, Mindray Z5) was used, equipped with a linear and a micro-convex transducer, with frequencies between 6.5-8.5 MHz. The type of transducer, as well as the frequency used, were chosen at the time of examination, depending on the gestational phase and structure to be assessed. To improve the transmission of ultrasound waves, ultrasound gel was used on the skin of the region to be examined.

The females went through weekly ultrasound evaluations, starting between 15-30 days after the date of the latest mating, until the day of delivery, totaling between 3-6 exams per female. All analyses were performed by the same experienced ultrasonographer and images with the measurements were later evaluated by a second examiner.

**Figure 1:**
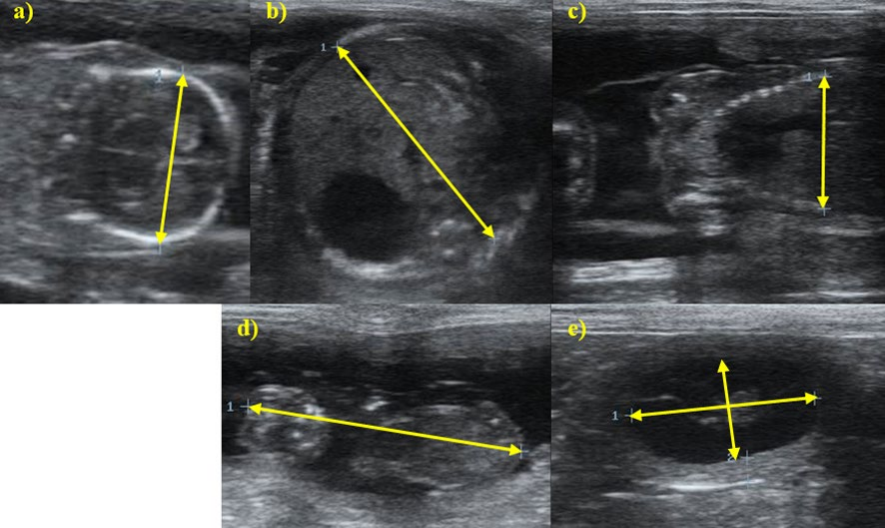
Ultrasound images of the measurements taken in fetal and non-fetal structures in pregnant Chihuahua female dogs. (a) ultrasound measurement of the biparietal diameter – canine fetus of the Chihuahua breed 16 days before delivery; (b) ultrasound measurement of abdominal diameter – canine fetus of the Chihuahua breed six days before delivery; (c) ultrasonographic measurement of the thoracic diameter – canine fetus of the Chihuahua breed 16 days before delivery; (d) ultrasonographic measurement of the craniocaudal length – canine embryo of the Chihuahua breed 32 days before delivery; (e) ultrasonographic measurement of the diameter of the internal chorionic cavity – fetal gallbladder of the female Chihuahua 35 days before delivery. B-mode ultrasonography, equipment Mindray Z5, linear transducer, 7.5MHz.

The following fetal/embryonic and extra-fetal structures were assessed: biparietal diameter (BPD): obtained from the external measure of the greater distance between the parietal bones, in the transverse view of the skull (Son et al., 2001; Groppetti et al., 2015). Abdominal Diameter (ABD): measured using the section of the abdomen at the largest diameter at stomach’s height (Son et al., 2001). Thoracic diameter (TD): measurement performed on the greatest distance from the cross section of the chest, at the point of the last rib (Jabin et al., 2007). Craniocaudal length (CCL): obtained by the measurement of length from the most cranial point on the skull to the caudal edge to the perineum (Son et al., 2001; Beccaglia and Luvoni, 2006). Inner diameter of the chorionic cavity (IDCC): corresponding to the perpendicular distances between the inner walls of the chorionic cavity, in its largest and smallest diameters, determined by taking the mean of those two ICC diameters made at 90° angles from one side of the trophoblastic decidual reaction to the other (Beccaglia and Luvoni, 2006; Groppetti et al., 2015) (Figure 1).

The data obtained from the measurements (BPD, ABD, TD, CCL and IDCC) were presented as averages of all fetuses examined during the evaluation of each bitch and were retroactively related to the remaining days before parturition (DBP), from the date that labor occurred as reported by the breeder, considered day zero. We performed a simple linear regression and an ANOVA analysis, using the program IBM SPSS Statistics (IBM Corp. Released 2017. IBM SPSS Statistics for Windows, Version 25.0. IBM Corp), using the equation *y* = a*x*+b, where “*y*” corresponds to the days before delivery, “*x*” is the measurement of the structure, “a” is the constant coefficient and “b” the first order coefficient.

## Results

Of the thirteen monitored pregnancies, 43 living puppies were born, all from natural and a term delivery, no cases of fetal mummification or stillborns. The size of the litters ranged from 1-7 puppies, with an average of 3.2 offspring per female. In total, 64 ultrasound examinations were performed, and on each assessment, it was possible to individually evaluate until five fetuses.

It was possible to collect the measurements between the 44rd day prior to delivery and the day of delivery, and all evaluated parameters showed a significant correlation (*P*<0.05) to the gestational age, demonstrating that the fetal parameters of the BPD, TD, ICCD and ABD have higher correlation with the days until delivery than the craniocaudal length, as detailed in Table 1.

**Table 1:** Gestational parameters (*X*), the minimum and maximum values of the averages of each measurement, the interval of days before parturition (DBP), linear regression equation of days until delivery (*Y*), correlation coefficient (*r*), coefficient of determination (*R^2^*) and estimated standard deviation (SD) corresponding to the structures assessed by B mode ultrasonography in thirteen pregnant Chihuahua females.

**Gestational Parameter (*X*)**	**Minimum and maximum values (cm)**	**DBP**	***Y***	***r***	***R^2^***	**SD**	**p-value**
IDCC (*X1*)	0.43-3.08	44-23	-7.51X1 + 46.09	0.96	0.93	1.76	<0.0001
CCL (*X2*)	0.83-4.50	34-20	-2.63X2 + 34.51	0.87	0.75	2.00	<0.0001
BPD (*X3*)	0.91-2.48	25-0	-15.46X3 + 38.72	0.99	0.98	1.21	<0.0001
TD (*X4*)	0.86-3.43	25-0	-10.96X4 + 33.50	0.96	0.92	2.18	<0.0001
ABD (*X5*)	1.10-4.10	25-0	-8.61X5 + 35.28	0.95	0.89	2.55	<0.0001

Fetal/embryonic and extra-fetal structures assessed by ultrasound: inner diameter of the chorionic cavity (IDCC); craniocaudal length (CCL); biparietal diameter (BPD); thoracic diameter (TD) and abdominal diameter (ABD).

It can be seen that the equations generated by the linear regression of the different measures studied were statistically validated (P <0.0001). In addition, it was observed that all equations had high coefficient of determination (R^2^), which represents the high degrees of reliability of the formulas. The biparietal diameter measure (BPD) showed the highest significant correlation (r = 0.99, P <0.0001) with the days until delivery (DBP). For this measure, it was also observed that the generated formula (y = -15.46X3 + 38.72) presented the highest determination factor with R^2^ = 0.98 (P <0.0001), which means that through it one can explain 98% of the DBP events in the Chihuahua bitches. The other 2% is the statistical error, which is probably included in the standard deviation (SD) generated by the formula, of ± 1.21 days.

**Figure 2:**
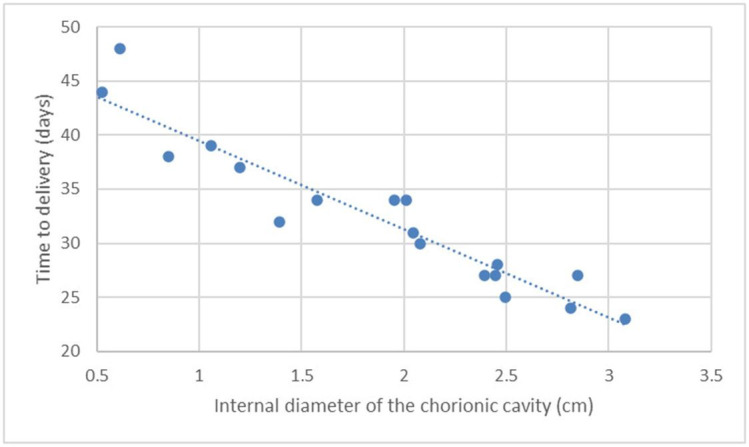
Scatter chart and regression line of the function of days before parturition (DBP; *Y*) in relation to the ultrasonographic measurement of the diameter of the internal chorionic cavity (ICCD; *X1*) on canine fetuses of the Chihuahua breed.

The variables showed a linear distribution, increasing with the development of the pregnancy and consequent decrease in the number of days until delivery. Scatter charts were drawn up with their respective functions of linear regression by correlating the variable with the DBP (Figures 2 [Fig gf03] [Fig gf04] [Fig gf05]-6).

**Figure 3 gf03:**
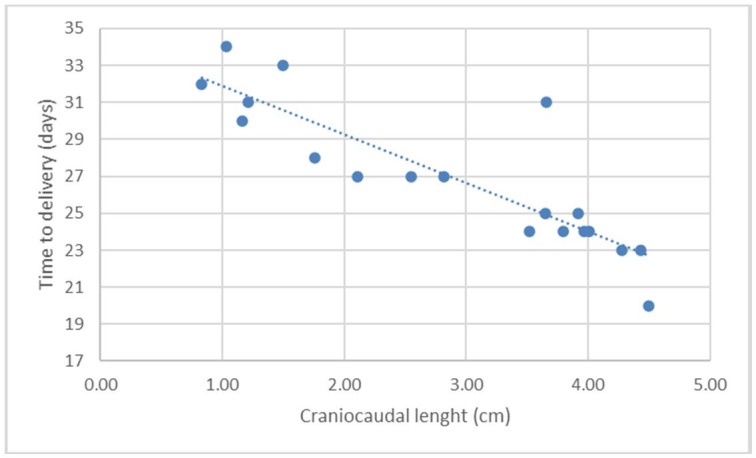
Scatter chart and regression line of the function of days before parturition (DBP; *Y*) in relation to the ultrasonographic measurement of the craniocaudal length (CCL; *X2*) on canine fetuses of the Chihuahua breed.

**Figure 4 gf04:**
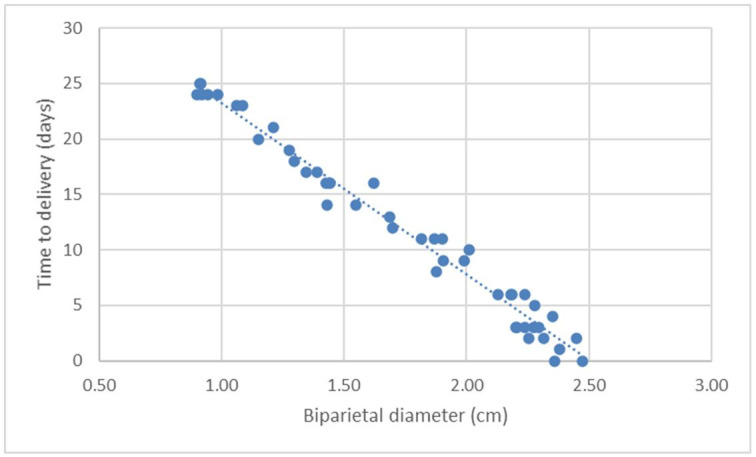
Scatter chart and regression line of the function of days before parturition (DBP; *Y*) in relation to the ultrasonographic measurement of the biparietal diameter (BPD; *X3*) on canine fetuses of the Chihuahua breed.

**Figure 5 gf05:**
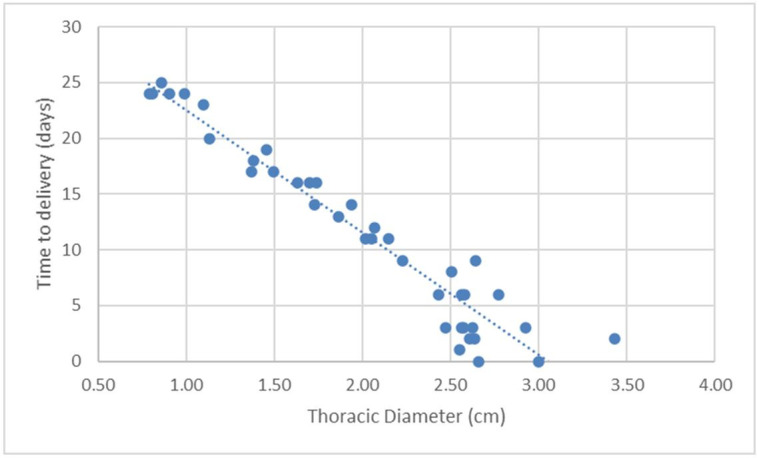
Scatter chart and regression line of the function of days before parturition (DBP; *Y*) in relation to the ultrasonographic measurement of the thoracic diameter (TD; *X4*) on canine fetuses of the Chihuahua breed.

**Figure 6:**
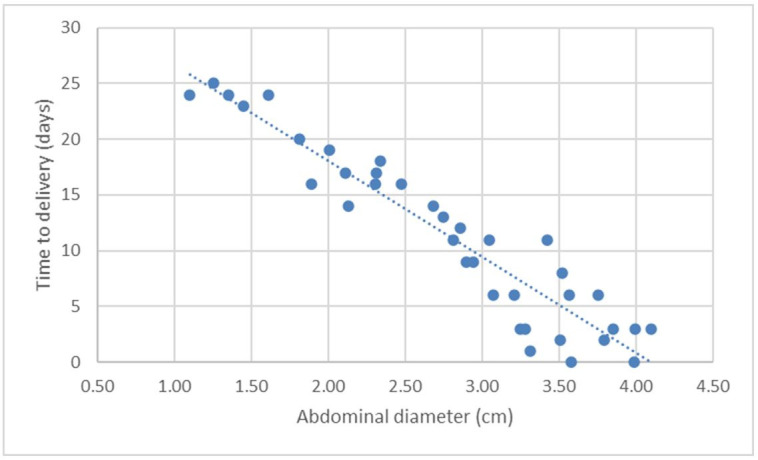
Scatter chart and regression line of the function of days before parturition (DBP; *Y*) in relation to the ultrasonographic measurement of the abdominal diameter (DAB; *X5*) on canine fetuses of the Chihuahua breed.

## Discussion

Due to the difficulties mentioned earlier in estimating the exact date of conception in female dogs, without hormonal dosages or vaginal cytology, to estimate the gestational age based only on the date of mating is quite inconsistent (Luvoni and Grioni, 2000). Therefore, the study was designed to estimate the number of days until delivery. Several studies have found highly-significant correlations between the fetal measurements and the number of days until delivery in specific breeds of dogs of varying sizes, however, none of these studies have solely studied the Chihuahua breed (Melo et al., 2006; Silva et al., 2007; Jabin et al., 2007; Teixeira et al., 2009; Camargo et al., 2011; Groppetti et al., 2015; Cecchetto et al., 2017; Socha and Janowski, 2017).

This study found a strong positive correlation between the biparietal (*r =* 0.99, *P<*0.05), thoracic (*r =* 0.96, *P<*0.05), inner chorionic cavity (*r* = 0.96, P<0.05) and abdominal (*r =* 0.95, *P<*0.05) diameters with the remaining DBP in female Chihuahuas. In several studies, BPD was identified as the measurement with a stronger correlation with the gestational period, from the second half of pregnancy in dogs of different breeds. It was considered a reliable measurement and easy to obtain (Luvoni and Grioni, 2000; Melo et al., 2006; Jabin et al., 2007; Silva et al., 2007; Cecchetto et al., 2017).

Socha and Janowski (2018) found a correlation of 0.94 (*P<*0.05) of the BPD with the DBP in miniature dog breeds. The author suggests that formulas used for small and medium-sized breeds, such as those described by Luvoni and Grioni (2000) might be used in female dogs of miniature breeds. The regression formula (*y* = 1.6190 × BPD - 39.70) presented in the study (Socha and Janowski, 2018) differs from the formula presented in this research and features a large variation of the values found for the Chihuahua, which highlights the need to study this breed individually and to standardize an adequate calculation.

In the investigation by Son et al. (2001), when studying the correlation between gestational age and date of delivery with the gestational parameters of two miniature breeds (Yorkshire Terrier and Maltese), a strong correlation was found (*r* = 0.99) between the measurements of the inner chorionic cavity diameter (ICCD) and the gestational age in the period between 20-37 days of pregnancy. Similarly to Jabin et al. (2007), who calculated the volume of the chorionic vesicle, based on the ICCD, and also identified a high correlation with the gestational age (*r =* 0.96) in female Yorkshire Terriers. In the present study, it was observed a strong correlation (*r =* 0.96, *P<*0.05) between the ICCD and the DBP in the period between 44-27 days before delivery, presenting the ICCD as the best parameter to predict the delivery in the early half of pregnancy.

Also, in the study by Jabin et al. (2007), the CCL showed a correlation coefficient of 0.97 with the gestational age, when measured between 21-27 days until 42 days of pregnancy, which is different from this study, where a weaker correlation (0.87) was found when measuring that fetal parameter between 34-20 days before delivery. This small window of fourteen days of pregnancy to perform the measurement provided little data and, for some females, only one measurement was taken, which limits the clinical application. This is because, when fetuses grow, it bends, altering the values of this measurement. In addition, when pregnancy progresses, the transducer used in the experiment was not capable of forming an image of the entire fetal length, preventing the measurement in more advanced pregnancies.

In this study the coefficients of determination found for the equations generated with the measurements made at the end of pregnancy (BPD, TD and ABD) were always high (0.98, 0.92 and 0.89, respectively), which represents an accurate prediction of delivery. However, in advanced pregnancies, for greater accuracy in predicting the delivery onset and diagnosis of fetal distress, it is important to correlate the values found in biometric formulas with other ultrasound analyses such as organogenesis (Pieri et al., 2015) and measurement of heart rate fetal (Gil et al., 2014), or more complex studies, through Doppler ultrasound, to assess the resistivity of the umbilical artery (Giannico et al., 2015). Such assessments, associated with clinical signs, like rectal temperature, provide greater security for the professional to monitor the delivery and, especially, schedule a cesarean section.

All the female dogs experienced natural deliveries; only two dogs were assisted by a veterinary medical team, and there was no incidence of dystocia in the studied population. In some previous studies, high rates of labor dystocia were observed in the Chihuahua breed (Gaudet, 1985; Bergström et al., 2006; O’Neill et al., 2017), considered a dog of large cranial dimensions, that might need an episiotomy due to the head of the puppy being entrapped in the vulvar opening (Traas, 2008).

In the study by O’Neill et al. (2017), while assessing more than 18 thousand female dogs, the Chihuahua breed presented the highest number of cases of dystocia and was the fourth highest in terms of prevalence and risk factors for dystocia, immediately after three brachycephalic breeds (French Bulldog, Boston Terrier, and Pug). As the records presented in the literature are from other countries, it is possible that the genetic selection of the Brazilian commercial kennels made it possible to reduce this incidence, as no events of cesarean section or episiotomy have been recorded in Chihuahuas in recent years in the clinical routine of the hospital where the study was developed, besides the fact that this is not such a common breed in Brazil as it is in other countries.

The predisposition of the Chihuahua breed to have a dystocia justifies the need to monitor the gestational age of these females and to estimate the probable date of delivery, to prepare both the guardian and the veterinary team for the possibility of a cesarean section or assisted delivery. By knowing the date of delivery, it is possible to be attentive to any abnormal behavior, to take measures to anticipate problems that may cause fetal death or put the life of the dam at risk, or to decide to perform an elective cesarean section (Socha and Janowski, 2018).

## Conclusion

The Chihuahua breed has peculiarities in the fetal biometry, making it necessary to standardize fetal measurement calculations by ultrasonography, for the better pregnancy and parturition management. The high correlations observed between the embryo-fetal measures and the remaining time until delivery, added to high factors of determination obtained by specific formulas established, especially with the BPD use, allow an accurate prediction of the date of birth in this breed.
